# A rapid and non-destructive method for spatial–temporal quantification of colonization by *Pseudomonas syringae* pv. *tomato* DC3000 in Arabidopsis and tomato

**DOI:** 10.1186/s13007-021-00826-2

**Published:** 2021-12-13

**Authors:** Leonardo Furci, David Pascual-Pardo, Jurriaan Ton

**Affiliations:** 1 School of Biosciences, University of Sheffield, Western Bank, Sheffield, UK; 2 P3 Centre for Plant & Soil Biology, Institute for Sustainable Food, University of Sheffield, Sheffield, UK; 3 Plant Epigenetics Unit, Okinawa Institute of Science and Technology, Onna-son, Okinawa, Japan

**Keywords:** Arabidopsis, Tomato, *Pseudomonas syringae* pv. *tomato*, Bioluminescence, Non-destructive assay, Spatial–temporal pathogen colonisation

## Abstract

**Background:**

The bacterial leaf pathogen *Pseudomonas syringae* pv *tomato* (*Pst*) is the most popular model pathogen for plant pathology research. Previous methods to study the plant-*Pst* interactions rely on destructive quantification of *Pst* colonisation, which can be labour- and time-consuming and does not allow for spatial–temporal monitoring of the bacterial colonisation. Here, we describe a rapid and non-destructive method to quantify and visualise spatial–temporal colonisation by *Pst* in intact leaves of Arabidopsis and tomato.

**Results:**

The method presented here uses a bioluminescent *Pst* DC3000 strain that constitutively expresses the *luxCDABE* operon from *Photorhabdus luminescens* (*Pst::LUX*) and requires a common gel documentation (Gel Doc) system with a sensitive CCD/CMOS camera and imaging software (Photoshop or Image J). By capturing bright field and bioluminescence images from *Pst*::*LUX*-infected leaves, we imaged the spatiotemporal dynamics of *Pst* infection. Analysis of bioluminescence from live *Pst* bacteria over a 5-day time course after spray inoculation of Arabidopsis revealed transition of the bacterial presence from the older leaves to the younger leaves and apical meristem. Colonisation by *Pst:LUX* bioluminescence was obtained from digital photos by calculating relative bioluminescence values, which is adjusted for bioluminescence intensity and normalised by leaf surface. This method detected statistically significant differences in *Pst::LUX* colonisation between Arabidopsis genotypes varying in basal resistance, as well as statistically significant reductions in *Pst::LUX* colonisation by resistance-inducing treatments in both Arabidopsis and tomato. Comparison of relative bioluminescence values to conventional colony counting on selective agar medium revealed a statistically significant correlation, which was reproducible between different Gel Doc systems.

**Conclusions:**

We present a non-destructive method to quantify colonisation by bioluminescent *Pst::LUX* in plants. Using a common Gel Doc system and imaging software, our method requires less time and labour than conventional methods that are based on destructive sampling of infected leaf material. Furthermore, in contrast to conventional strategies, our method provides additional information about the spatial–temporal patterns of *Pst* colonisation.

**Supplementary Information:**

The online version contains supplementary material available at 10.1186/s13007-021-00826-2.

## Background

*Pseudomonas syringae* is a hemi-biotrophic Gram-negative bacterial species that infects a wide range of plant species [1]. In the early 1990s, the *P. syringae* pathovar *tomato* strain DC3000 (*Pst*) from tomato was found to infect various accessions of *Arabidopsis thaliana* (Arabidopsis), which led to the establishment of the most popular pathosystem in phytopathological research [2]. The systematic study of the *Pst*-Arabidopsis interaction has yielded crucial discoveries about plant immunity and plant-pathogen interactions, including bacterial virulence [3], resistance (R)-genes and effector-triggered immunity [4, 5], perception of microbial associated molecular patterns (MAMPs) [6, 7], basal resistance [8–10], and various forms of acquired resistance [11–14].

Irrespective of the nature of the research, accurate quantification of pathogen colonisation is crucial for the determination of host resistance. For the *Pst*-Arabidopsis system, the gold standard to quantify bacterial colonisation is based on counting colony forming units (CFUs) on selective agar plates of serial dilutions from homogenised *Pst*-inoculated leaves [4, 15]. Although the use of microtiter plates and multichannel pipettes has made this method more efficient [15], the homogenisation of leaf tissue and subsequent dilution plating remains a labour-intensive step that is prone to errors. The availability of transgenic bioluminescent *Pst* DC3000 strain expressing a stable chromosomal insertion of the *luxCDABE* operon from the insect pathogen *Photorhabdus luminescens* into *Pst* (henceforth, *Pst::LUX*) has enabled an alternative method for determining *Pst* colonization that does not rely on dilution plating [16]. This procedure relies on measuring photon emission by living *Pst::LUX* cells in excised leaf discs or ground suspensions from infected plants. Nonetheless, the quantification of bioluminescence with a luminometer still relies on destructive sampling and does not provide information about the spatial–temporal colonization by *Pst*.

In this study, we describe an improved method for the quantification of *Pst* colonisation in plants, which requires a common gel documentation (Gel Doc) system with a high-sensitivity CCD/CMOS camera that is suitable for quantification of chemiluminescence. There are two main advantages to this method compared to previously described methods of *Pst* quantification. Firstly, our method is non-destructive, allowing for repeated measures on the same sample/plant over a time-course without having to increase population size. Secondly, the image-based data acquisition from infected leaf tissues captures valuable information about the spatial–temporal patterns of bacterial colonization in intact plants.

## Materials and methods

### Plant materials and growth conditions

Arabidopsis seeds from wild-type (accession Col-0) and hypersusceptible *NahG* (Col-0; [17]) were stratified in water at 4 °C in the dark for 4 days. Seeds were then sown in a sand:M3 compost mixture (1:3) and cultivated under short-day conditions for 2.5 weeks (8.5 h light/15.5 h dark, 21 °C, 60% relative humidity, ~ 125 µmol s^−1^ m^−1^ light intensity). A total of 24 plants of each genotype/treatment combination were grown in three 60 ml-pots (8 plants/pot). Tomato seeds (cv. MoneyMaker) were planted in M3 compost and cultivated under long-day conditions for 2 weeks (16 h light/8 h dark, 25 °C, 60% relative humidity, ~ 200 µmol s^−1^ m^−1^ light intensity). A total to 15 tomato plants per treatment were grown in individual 100 ml-pots. Resistance was induced at 2 days before bacterial inoculation. For induction of resistance, Arabidopsis plants were sprayed with 250 μM benzothiadiazole (BTH) as Bion^®^ (Syngenta) suspended in reverse-osmosis water, whereas tomato plants were sprayed with 2.5 mM salicylic acid (SA) suspended in reverse-osmosis water.

### Bacteria preparation and inoculation

Before each inoculation, transgenic *Pst::LUX* bacteria [16] were cultured from a frozen stock (− 80 °C; 20% v/v glycerol) of an overnight (O/N) culture from single colony. One day before inoculation, 1 ml of frozen stock was added to 50 ml of KB medium (prepared from 1L: 20 g Proteose Peptone; 15 ml glycerol; 1.5 g K_2_HPO_4_; 1.5 g MgSO_4_.7H_2_O; final pH 7.2) containing 50 μg/ml Rifampicin and 50 μg/ml Kanamycin for positive selection of transgenic *Pst::LUX* cells. Bacteria were grown O/N at 28 °C in a shaking incubator (Grant-Bio; ES-20). O/N cultures of *Pst::LUX* were centrifuged at 3000 rpm for 3 min, after which the pellet was re-suspended in 10 mM MgSO_4_ to a final density of 0.2 OD_600_, supplemented with 0.01% v/v surfactant (Silwet L-77, Lehle Seeds), and sprayed onto the leaf surface of 2.5-week-old Arabidopsis plants or 2-week-old tomato. Several hours before inoculation and subsequent days after inoculation, plants were kept at 100% relative humidity (RH) to facilitate infection.

### Non-destructive quantification of *Pst* colonisation by bioluminescence

At different days post inoculation (dpi), pots were placed in the dark room of a G:BOX Chemi XRQ (GeneSys) Gel Doc system. Bright field images of infected plants were taken, using the G:BOX Chemi XRQ internal LED illumination (80 ms exposure time). After subsequent incubation in complete darkness of at least 1 min., images of bacterial bioluminescence were acquired without moving the pots (90 s. exposure time, maximum iris opening). To assess robustness of the method, an independent Arabidopsis experiment was conducted with an alternative Gel Doc system (ChemiDoc XRS + Imager, BioRad), using 50 ms. exposure time for bright field images and 60 s. exposure time for bioluminescence acquisition. The resolution of the images acquired for bioluminescence analysis was 2300 × 1700px for the G:BOX Chemi XRQ Gel Doc system and 3100 × 2300px for the ChemiDoc XRS + Imager, allowing us to image multiple pots at once over a total surface area of 1039 cm^2^ and 540 cm^2^, respectively.

Image-based quantification of bioluminescence was carried out using the common imaging software Photoshop CS6 (Adobe). For each plant, the outlines of the leaves were obtained from bright field images, using a combination of the “Magic Wand” and “Lasso” selection tools. These selections were then saved (“Save Selection” function) and transposed onto the bioluminescence images (“Load Selection” function). The bioluminescence signal from infected leaves were obtained from unprocessed bioluminescence image files (exported as TIFF files) using the “Histogram” function in Adobe Photoshop, which quantifies bioluminescence intensity as a function of pixel brightness (ranging from 0, black pixels, to 255, brightest white pixels) on the basis of the following formula:

$${\text{Relative bioluminescence}} = \frac{{\mathop \sum \nolimits_{i = 0}^{255} n_{i} \left( i \right)}}{{n_{tot} }}$$

where *n*_*i*_ indicates the number of pixels at each brightness *i* (0 to 255) within the selected area and *n*_*tot*_ indicates the total number of pixels corresponding to plant area, providing a relative bioluminescence metric that corrects for variation in plant size. To further correct for varying background levels, a concentric circle was placed around individual plants (~ 2 × rosette diameter). Background levels of luminescence were then calculated in the area outside the selected plant surface area, which was then subtracted from the relative bioluminescence value originating from within the plant surface area. Other open-source image analysis software, such as GNU-based GIMP or Java-based ImageJ, use an identical function to calculate this value. Because name and functionality of proprietary tools differ across software, we detailed the different steps required to replicate the analysis in each of these programs (Additional file 1: Fig. S1). For correlation analysis between non-destructive quantification of *Pst* colonisation by bioluminescence and destructive quantification by dilution plating, Arabidopsis plants from each genotype/pre-treatment combination were numbered during image quantification, after which leaf material was harvested for colony plating.

### Destructive quantification of *Pst* colonisation by colony plating

Following image acquisition, each Arabidopsis seedling was weighted and ground in 1.5 ml Eppendorf tubes containing 750 μl of ice-cold 10 mM MgSO_4_, using a pellet pestle. From each homogenised suspension, an aliquote of 50 µl was then serial-diluted, using 96-wells microtiter plates (Costar^®^) containing 200 µl of 10 mM MgSO_4_. Twelve samples in each plate were serial-diluted eight times (fivefold dilution steps) and plated onto selective KB agar plates containing 50 μg/ml Rifampicin and 50 μg/ml Kanamycin, using 96-wells Scienceware^®^ replicator (Sigma-Aldrich, plating 10 µl per pin). From each well, two technical replicates were plated onto separate plates and incubated at 28 °C incubator for 36 h before enumeration of colony forming units (CFUs). For each sample, CFU counts were averaged between two-to-three different serial dilutions and between technical replicates and normalised to plant fresh weight (mg).

### Statistical analysis

One-way analysis of variance (ANOVA) with Tukey’s post-hoc test and Student’s t-tests were performed using SPSS Statistics (v24.0, IBM). Pearson’s correlation analysis was performed using R software (v4.1.0).

## Results

### Quantification of in planta bioluminescence by *Pst::LUX*

**Fig. 1:**
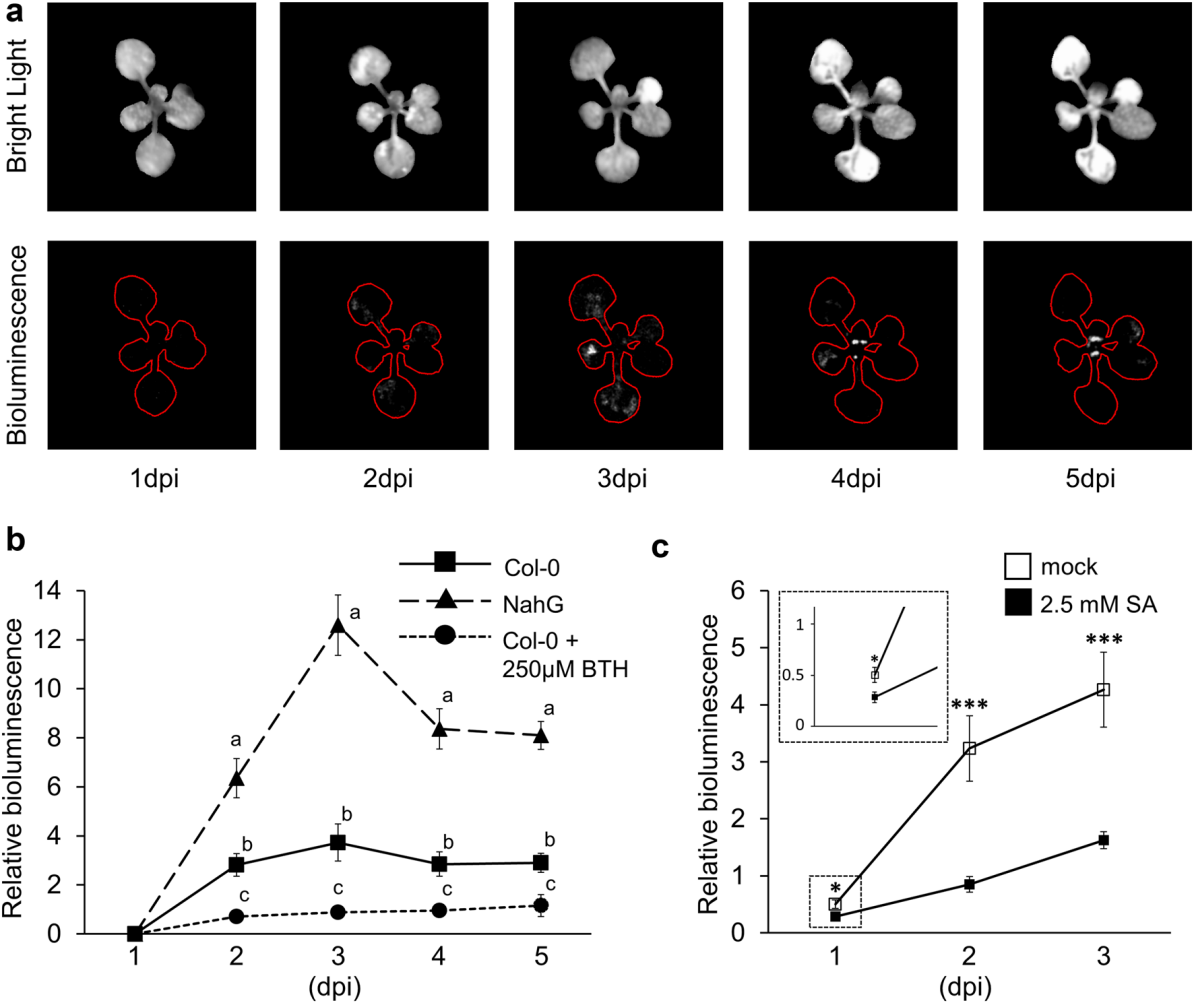
Non-destructive visualisation and quantification of leaf colonisation by bioluminscent *P. syringae* pv. *tomato* D3000 expressing the *luxCDABE* operon from *Photorhabdus luminescens* (*Pst::LUX*). **A** Representative example of the spatial–temporal pattern *Pst::LUX* colonisation in a hyper-susceptible *NahG *plant of Arabidopsis. Top panels show black and white images of the plant taken under bright field illumination. Bottom panels show bacterial bioluminescence acquired from the same plant by a quantum efficiency CCD camera in complete darkness. Red outlines indicate the plant surface area obtained from the bright field images. **B** Quantification of *Pst::LUX* colonisation of Arabidopsis plants that vary in salicylic acid (SA)-dependent resistance. Bacterial bioluminescence was measured in hyper-susceptible *NahG*, wild-type Col-0 and Col-0 pretreated with 250 μM of the resistance-inducing SA analogue BTH. Data represent relative bioluminscence values normalised by leaf surface area per plant. Statistically significant differences between genotypes/treatments at each time-point (letters) were assesed by one-way ANOVA, followed by Tukey’s post-hoc for multiple comparisons (*p* < 0.05). Error bars represent standard errors of the mean (*n* = 24). **C** Quantification of *Pst::LUX* colonisation of tomato plants (cv. MoneyMaker) that vary in SA-dependent resistance. Bioluminescence was measured in plants pre-treated with water (mock; susceptible) or 2.5 mM SA (resistant). Data represent relative bioluminscence values normalised by leaf surface area per plant. Statistically significant differences between treatments at each time-point were assesed by a Student’s *t*-test (*p < 0.05; ***p < 0.001). Error bars represent standard error of the mean (*n* = 15). dpi: days post inoculation

The method described here relies on a Gel Doc system fitted with CCD/CMOS camera for detection of chemiluminescence and the transgenic *Pst* DC3000 strain, which carries a stable chromosomal insertion of the *luxCDABE* operon from *P. luminescens* and is kanamycin-resistant [16]. Following the acquisition of digital bright field images of inoculated plants, the bacterial bioluminescence signal is acquired in complete darkness, without moving the pots. Before acquisition of the bioluminescence signal by the camera, it is important keep plants in complete darkness for at least 1 min. to prevent delayed chlorophyll fluorescence, which is partially within the visible spectrum and can thus be detected by camera as a confounding signal [18]. To prevent variable contrast-enhancing modifications by proprietary software of the Gel Doc system, the unprocessed bioluminescence images (TIFF) should be exported before selection and quantification of bioluminescence by imaging software. Bright field and bioluminescence should be of identical pixel size to facilitate the superimposing of the bioluminescence signal onto leaf area from the bright field image. Examples of bright field and bioluminescence images of Arabidopsis are presented in Fig. 1a and Additional file 2: Fig. S2, showing that the bacteria migrate from fully expanded leaves at 2 and 3 dpi to younger leaves in the centre of the rosette by 4 and 5 dpi (Fig. 1a and Additional file 2: Fig. S2). Hence, non-destructive analysis of bioluminescence by *Pst::LUX* visualises the spatial patterns of *in planta* colonisation by this pathogen.

### Time-course analysis of *Pst::LUX* colonisation in Arabidopsis plants vary in quantitative resistance

Using the method described above, we compared *Pst::LUX* colonisation after inoculation of 17-day-old Arabidopsis plants that vary in quantitative SA-dependent resistance (Fig. 1b). Transgenic *NahG* plants, which are strongly affected in endogenous accumulation of the defence regulatory hormone SA [17], displayed statistically enhanced levels of bioluminescence compared to Col-0 wild-type plants at all time-points analysed (Fig. 1b, one-way ANOVA). On the other hand, resistance-inducing treatment of Col-0 with 250 μM of the functional SA analogue BTH [11] at 2 days before bacterial inoculation resulted in a statistically significant reduction of bioluminescence compared to un-treated Col-0 plants (Fig. 1b; one-way ANOVA). Furthermore, the bioluminescence in hyper-susceptible *NahG* and un-treated susceptible Col-0 peaked at 3 dpi (Fig. 1b) Thus, our non-destructive quantification of *Pst::LUX* colonisation can distinguish differences in SA-dependent resistance over time.

### Time-course analysis of *Pst::LUX* colonisation in tomato

To examine whether our non-destructive method for quantification of *Pst::LUX* colonisation can be applied to other plant species than Arabidopsis, we measured bioluminescence in susceptible and resistant tomato plants at different days after inoculation. Since chemical activation of the SA response in tomato induces resistance against *Pst* [19], we treated leaves of 12-day-old tomato (cv. MoneyMaker) with 2.5 mM SA at 2 days before spray-inoculation with *Pst::LUX*. Bioluminescence was quantified from 1 to 3 dpi. At later time-points, plants started abscising diseased leaves, which prevented further reliable quantification of *Pst::LUX* colonisation. As shown in Fig. 1c, pre-treatment with SA resulted in a statistically significant reduction of relative bioluminescence compared to control-treated tomato across all time-points (Fig. 1c; Student’s *T*-test). Notably, our method was sufficiently sensitive to detect a small, yet statistically significant, difference as early as 1 dpi (Fig. 1c, inset). Hence, the non-destructive quantification of *Pst::LUX* colonisation is suitable to distinguish differences in SA-dependent resistance in multiple plant species across different stages of infection.

### Comparison of *Pst::LUX* quantification by bioluminescence and colony plating

**Fig. 2:**
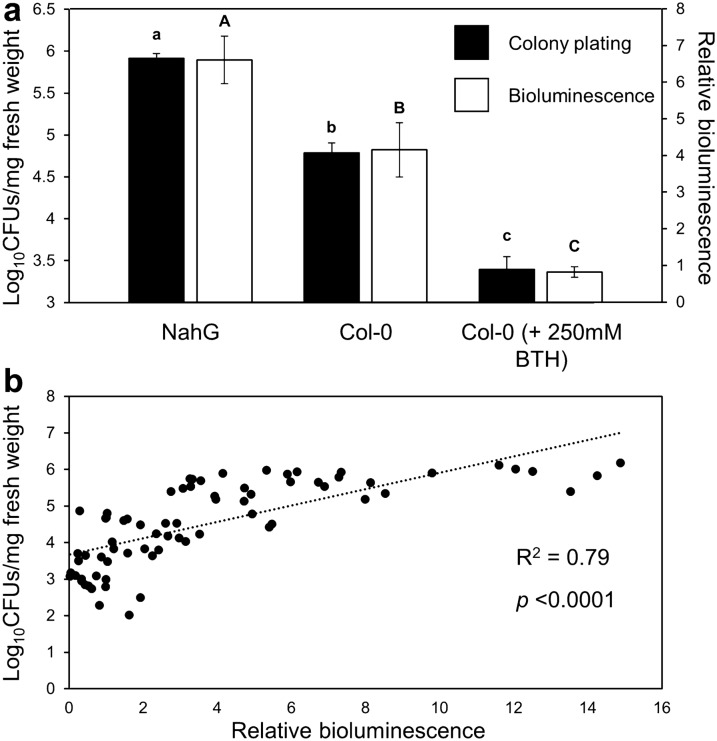
Comparison between methods for quantification of *Pst::LUX* colonisation in Arabidopsis plants varying in SA-dependent resistance. **A** Shown are mean Log_10_-transformed colony forming units (CFU) per milligram of fresh weight (black bars) and mean relative bioluminescence values per plant (white bars) for hyper-susceptible *NahG* plants, susceptible Col-0 plants and resistant Col-0 plants that had been pretreated with 250 μM BTH. Bacterial bioluminescence was measured *in planta* before samples were harvested for colony plating. Different letters indicate statistically significant differences between genotypes/treatments, using one-way ANOVA, followed by Tukey’s post-hoc analysis for multiple comparisons (*p* < 0.05; small letters: plate dilution analysis; capital letters: relative bioluminescence analysis). Error bars represent standard errors of the mean (*n* = 24). **B** Pearson’s correlation analysisb etween CFUs per milligram of plant fresh weight and relative bioluminescence. Dots represent individual samples from all genotypes/treatment combinations presented in **A**

To further validate the effectiveness of our method, we compared the non-destructive quantification of bioluminescence to conventional colony plating of *Pst::LUX* [15]. Quantification of colonisation by both methods was performed on the same plants at 3dpi after spray-inoculation, which is when bioluminescence peaked in the previous experiment (Fig. 1a, b). As is shown in Fig. 2a both methods detected similar and statistically significant differences in bacterial colonisation between hyper-susceptible *NahG*, Col-0, and Col-0 expressing BTH-induced resistance (Fig. 2a; one-way ANOVA followed by Tukey’s post-hoc test for multiple comparisons). Furthermore, Pearson’s correlation analysis between the relative bioluminescence values and fresh weight-normalised CFU counts revealed a positive correlation between both variables, which was statistically significant (Fig. 2b). In a separate experiment, we repeated the comparison between bioluminescence and colony plating for Col-0 and *NahG* plants with a different Gel Doc system (ChemiDoc XRS + Imager, BioRad), in order to assess robustness of the method with different lab equipment. Using a slightly shorter exposure time (60 s for the BioRad Gel Doc versus 90 s for the G:BOX Gel doc), we obtained similar results: a statistically significant difference in relatively bioluminescence between Col-0 and *NahG* (Additional file 3: Fig. S3a) and a positive correlation between relative bioluminescence and normalised CFU values that was statistically significant (Additional file 3: Fig. S3b, Pearson’s correlation, *p* = 0.0011). Based on both experiments, our method could detect variable levels of *Pst* colonisation at 3 dpi, ranging from as low as Log_10_(2.02) CFUs/mg of fresh plant weight (Fig. 2) to as high as Log_10_(10.39) CFUs/mg of fresh plant weight (Additional file 3: Fig. S3).

## Discussion

Precise quantification of plant colonisation by microbial pathogens is fundamental for mechanistic studies of plant-pathogen interactions. The *Pst*-Arabidopsis interaction is the most popular pathosystem in molecular plant pathology and has largely relied on colony (CFU) counting of infected leaf extracts after dilution plating on selective agar medium. While this method has been optimised for higher throughput [15], the homogenisation of plant tissues and dilution of samples remains a limitation. A previously described method, which measures bioluminescence emission by transgenic *Pst::LUX* bacteria in excised leaf discs of infected plants, has improved the throughput capacity and generated results that were comparable to colony plating [16]. However, this latter method remains destructive and therefore prevents analysis of the spatial–temporal patterning of pathogen infection, which is relevant for studies of quantitative resistance and pathogen effectors mediating systemic susceptibility. Here, we have introduced a novel non-destructive method that is based on bioluminescence by *Pst::LUX*, which combines high-throughput quantification with spatial–temporal visualisation of bacterial colonisation.

Our method requires a high-sensitivity CCD/CMOS camera, which are commonly used in Gel Doc systems to quantify chemiluminescence of non-radioactive probes, and common imaging software. For the calculation of relative bioluminescence, we used Adobe Photoshop, but open-source software such as ImageJ and GIMP are equally suitable. The non-destructive nature of our method allows for repeated measurements of the same plants. Consequently, time-course analysis of *Pst* colonisation is not confounded by inter-plant variation and does not require growing multiple sets of plants for each time-point. As a result, this method requires fewer plants, less controlled environment space, less time and less labour to generate precise time-course analysis of virulence and/or basal resistance in larger populations of plants. We have successfully used our method to quantify *Pst::LUX* colonisation in large-scale experiments consisting of > 700 samples in a single batch over a three-day time course, amounting to 2000 + individual measurements (Furci and Ton, unpublished results). By comparison, Fan et al. [16] reported scoring of bacterial bioluminescence in excised leaf discs consisting of 364 samples [16], whereas high-throughput colony plating is limited to approximately 100–150 samples per batch [15]. The method distinguishes subtle differences in quantitative resistance between plant genotypes or resistance-inducing treatments and yields comparable results to destructive colony plating (Figs. 1 and 2, Additional file 3: Fig. S3). Furthermore, we have successfully employed two commonly used Gel Doc systems (Fig. 2 and Additional file 3: Fig. S3) to quantify *Pst::LUX* colonisation in two different plant species (Fig. 1), which benchmarks our method for future studies of *Pst* pathosystems.

The image-based data collection of our method generates information about the spatiotemporal dynamics of *Pst* colonisation in individual plants (Fig. 1a and Additional file 2: Fig. S2). In Arabidopsis, Yu et al. [20] demonstrated that multiplication and vascular propagation of GFP-expressing *Pst* DC3000 is resisted through epigenetic mechanisms involving ROS1-dependent DNA de-methylation [20]. Furthermore, pathogenic *Pseudomonas syringae* isolates in other species have been reported to migrate from the initial site of inoculation through the vascular system [21, 22]. While other factors could explain the observed transition in bacterial colonisation from the mature leaves to younger leaves (e.g., a late onset of bacterial infection in the central rosette following spray-inoculation), the results presented in Figs. 1a and Additional file 2: Fig. S2 validate our methodology as a powerful tool to precisely quantify spatial–temporal colonisation of *Pst* with commonly available equipment. Future experiments involving local *Pst::LUX* inoculation followed by temporal analysis of relative bioluminescence could shed further lights on the systemic colonisation of *Pst* and associated defence mechanisms.

This ability to capture spatiotemporal patterning of *Pst* colonisation in a high-throughput manner creates new opportunities for large-scale genetic screens to identify bacterial effectors that facilitate plant colonisation or plant genes that control quantitative resistance barriers preventing systemic colonisation by the pathogen. Development of fully automated batch-processing pipelines of the bright field and bioluminescence images would further reduce the time required for the image analysis, which will be of particular benefit to large time-course experiments at population-wide scales (> 1000 samples). Previous studies have used changes in chlorophyll fluorescence to monitor *Pst* infection in a non-destructive manner [23]. Combining such non-destructive methods for the quantification of disease symptom development with our method would be a powerful tool to compare the molecular-genetic relationship between tolerance and resistance to *Pst*. Thus, our method can be linked to other phenotyping platforms, such as chlorophyll fluorescence imaging and hyperspectral imaging, which could drive further innovations in neural-network artificial intelligence software for automated data acquisition, integration and analysis [24, 25].

## Conclusions

We have developed a non-destructive method for rapid quantification and spatial–temporal visualisation of *Pst* colonisation in plants. Our method requires a commonly used Gel Doc system that is equipped with a sensitive CCD/CMOS camera for acquisition of chemiluminescence. The possibility of repeated measurements of *Pst* colonisation in the same plants reduces inter-plant variation in time-course analyses. The non-destructive and rapid nature of the method, combined with the image-based data acquisition, enables rapid quantitative screening of the spatiotemporal patterns of *Pst* colonisation, which opens up new avenues for large-scale genetic screens to identify bacterial virulence effectors and plant resistance barriers.

## Supplementary Information

**Additional file 1: Figure S1. Block-flow diagram detailing steps and tools required to perform bioluminescence analysis with different imaging software.**

**Additional file 2: Figure S2. Visualisation of** ***Pst::LUX*** **in susceptible** ***NahG*** **Arabidopsis plants**. Representative examples of the spatial–temporal pattern of *Pst::LUX* colonisation in two hyper-susceptible *NahG* plants of Arabidopsis. Top panels show black and white images of the same plant taken under bright field illumination. Bottom panels show bacterial bioluminescence acquired from the same plant by a quantum efficiency CCD camera in complete darkness. Red outlines indicate the plant surface area obtained from the bright field images. dpi: days post inoculation.

**Additional file 3: Figure S3. Comparison between methods for quantification of* Pst::LUX* colonisation in Arabidopsis genotypes varying in SA-dependent resistance, using a different Gel Doc system A)** Shown are mean Log_10_-transformed colony forming units (CFU) per milligram of fresh weight (black bars) and mean relative bioluminescence values per plant (white bars) for hyper-susceptible *NahG* plants and moderatly susceptible Col-0 plants. Bacterial bioluminescence was measured *in planta* before samples were harvested for colonoy plating. Asterisks indicate statistically sigificant differences between genotypes (Student’s t-test; ***: *p* < 0.001). Error bars represent standard errors of the mean (*n* = 12. **B)** Pearson’s correlation analysis between CFUs per milligram of plant fresh weight and relative bioluminescence. Dots represent individual samples from Col-0 and *NahG* genotypes presented in Fig. S3A.

## Data Availability

All raw data analysed in the current study and the *Pst::LUX* strain can be made available from the corresponding authors on reasonable request.
