# Supplementary figures and images for: A rapid and non-destructive method for spatial–temporal quantification of colonization by Pseudomonas syringae pv. tomato DC3000 in Arabidopsis and tomato
Source: Plant Methods. 2021 Dec 13;17:126. doi: 10.1186/s13007-021-00826-2 (PMC8667384; doi:10.1186/s13007-021-00826-2)

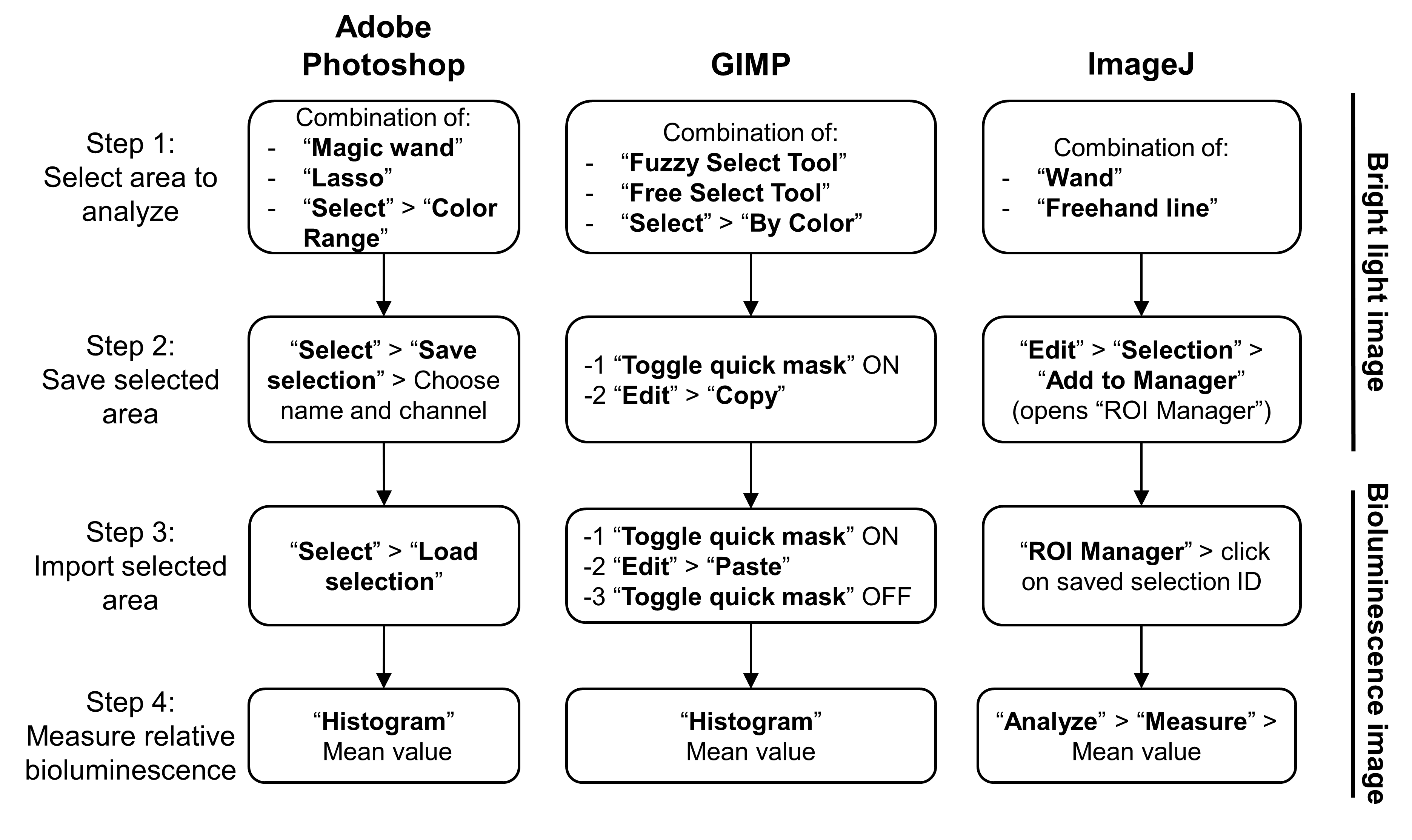

Supplement: Supplementary file 1 — Additional file 1: Figure S1. Block-flow diagram detailing steps and tools required to perform bioluminescence analysis with different imaging software. [file 13007_2021_826_MOESM1_ESM.tif]

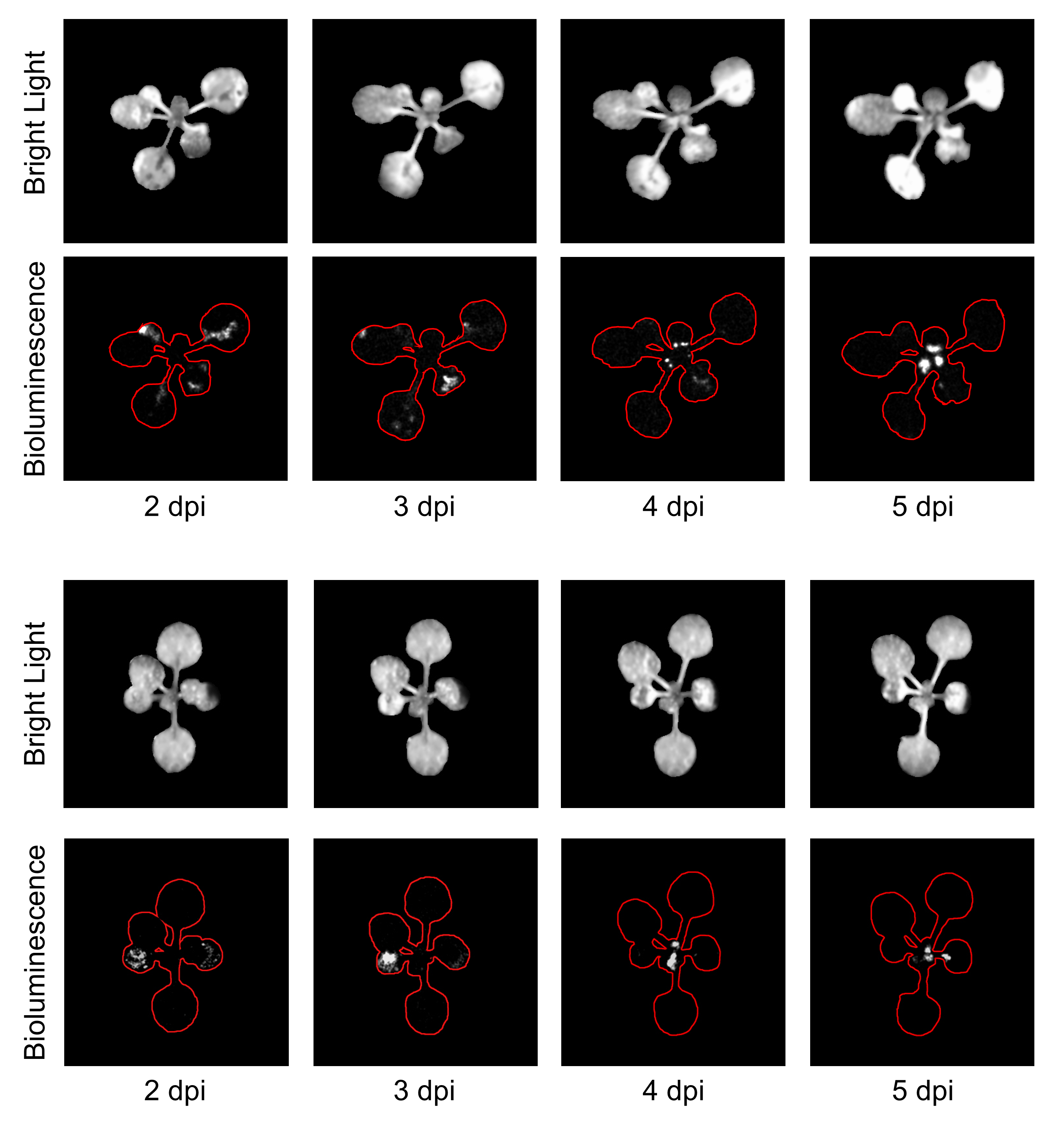

Supplement: Supplementary file 2 — Additional file 2: Figure S2. Visualisation of Pst::LUX in susceptible NahG Arabidopsis plants. Representative examples of the spatial–temporal pattern of Pst::LUX colonisation in two hyper-susceptible NahG plants of Arabidopsis. Top panels show black and white images of the same plant taken under bright field illumination. Bottom panels show bacterial bioluminescence acquired from the same plant by a quantum efficiency CCD camera in complete darkness. Red outlines indicate the plant surface area obtained from the bright field images. dpi: days post inoculation. [file 13007_2021_826_MOESM2_ESM.tif]

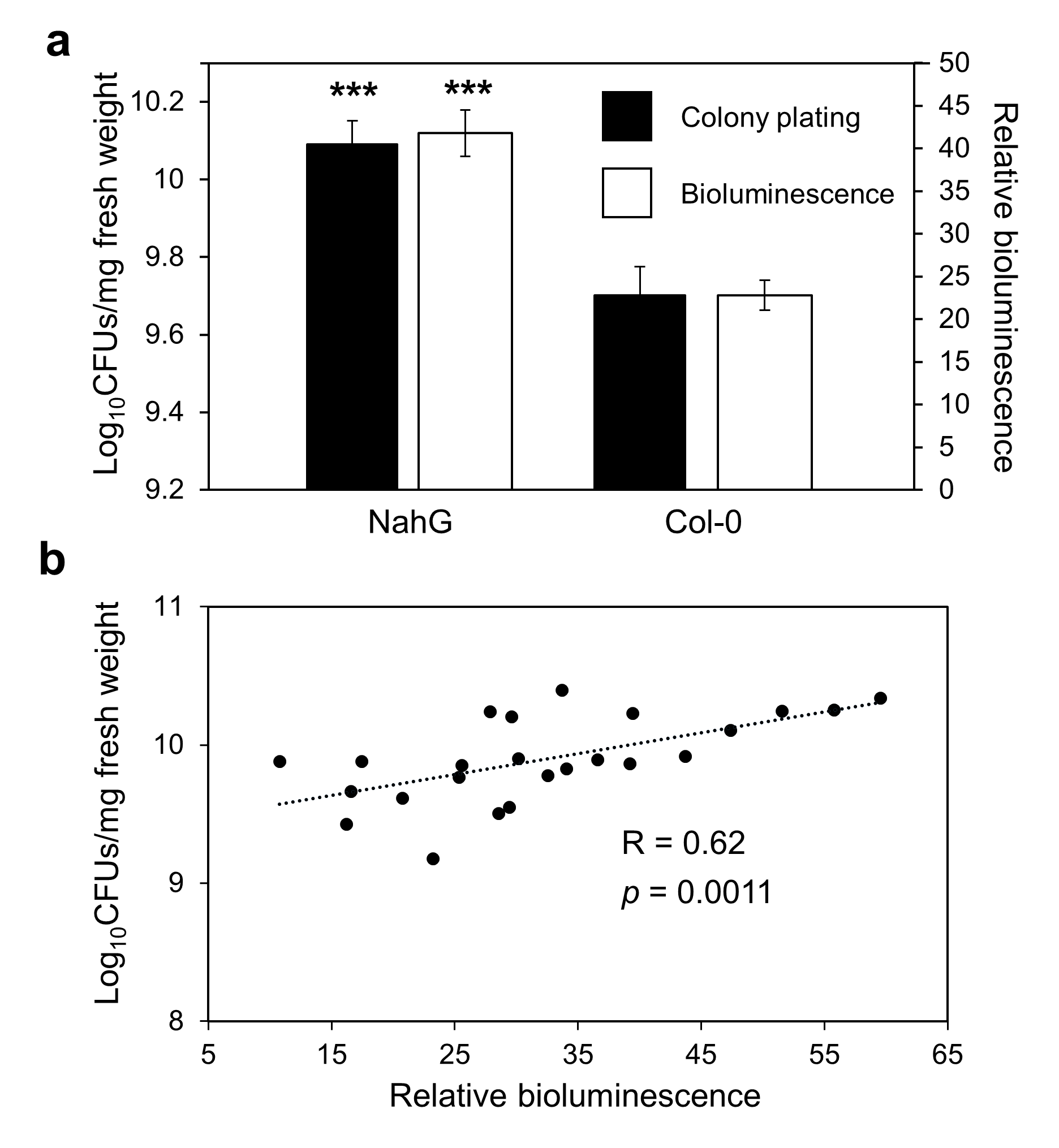

Supplement: Supplementary file 3 — Additional file 3: Figure S3. Comparison between methods for quantification of Pst::LUX colonisation in Arabidopsis genotypes varying in SA-dependent resistance, using a different Gel Doc system A) Shown are mean Log10-transformed colony forming units (CFU) per milligram of fresh weight (black bars) and mean relative bioluminescence values per plant (white bars) for hyper-susceptible NahG plants and moderatly susceptible Col-0 plants. Bacterial bioluminescence was measured in planta before samples were harvested for colonoy plating. Asterisks indicate statistically sigificant differences between genotypes (Student’s t-test; ***: p < 0.001). Error bars represent standard errors of the mean (n = 12. B) Pearson’s correlation analysis between CFUs per milligram of plant fresh weight and relative bioluminescence. Dots represent individual samples from Col-0 and NahG genotypes presented in Fig. S3A. [file 13007_2021_826_MOESM3_ESM.tif]
